# Association of environmental xylene exposure with diabetic kidney disease: a cross-sectional analysis and multi-omics evaluation of immune microenvironment mechanisms

**DOI:** 10.1080/0886022X.2026.2738974

**Published:** 2026-10-07

**Authors:** Yu Liu, Xinni Zhang, Jie Song, Tingwei Cheng, Lan-Gen Zhuang, Xiao-Yan Pei, Guo-Xi Jin

**Affiliations:** Department of Endocrinology, The First Affiliated Hospital of Bengbu Medical University, Bengbu, China

**Keywords:** Diabetic kidney disease, volatile organic compounds, xylene, macrophage polarization, single-cell RNA sequencing, immune microenvironment

## Abstract

Diabetic kidney disease (DKD) progression is driven by residual inflammation, potentially exacerbated by volatile organic compounds (VOCs). However, the impact of xylene exposure on the DKD immune microenvironment remains unclear. We evaluated the association between urinary VOC metabolites and DKD prevalence using NHANES data. Network toxicology and bulk transcriptomics (GSE142025) were integrated to identify core targets and profile immune infiltration. Single-cell RNA sequencing (GSE209781) validated macrophage heterogeneity and hub gene dynamics, and molecular docking assessed xylene-receptor binding affinities. Elevated urinary xylene metabolite (DPMA) correlated with increased DKD odds (OR = 4.03, 95% CI: 1.80–9.00). Multi-omics identified ITGAM, CSF1R, and CTSS as primary hub genes enriched in immune pathways. Both bulk and single-cell RNA sequencing analyses revealed that DKD tissues exhibited M2-like macrophage enrichment and an expansion of fibrotic SPP1+ subsets. This altered microenvironment positively correlated with hub gene upregulation along the macrophage developmental trajectory. Molecular docking suggested potential binding between xylene isomers and these receptors (binding free energies: −4.7 to −5.9 kcal/mol). Environmental xylene exposure is independently associated with higher DKD odds. These findings generate the hypothesis that xylene may interact with specific macrophage receptors (ITGAM, CSF1R, CTSS), potentially promoting M2-like macrophage polarization and fibrotic remodeling. This putative environmental-immunological axis warrants further experimental validation.

## 1. Introduction

Diabetic kidney disease (DKD) is recognized as one of the most prevalent microvascular complications of diabetes, currently ranking as the leading global cause of both chronic kidney disease (CKD) and end-stage renal disease (ESRD) [1,2]. In routine clinical practice, the management of DKD primarily relies on intensive glycemic control, blood pressure regulation *via* renin-angiotensin-aldosterone system (RAAS) blockade, and the application of sodium-glucose cotransporter-2 (SGLT2) inhibitors [3,4]. However, evidence from large-scale clinical cohorts indicates that even after optimizing metabolic and hemodynamic parameters, a substantial proportion of patients continue to experience renal function decline [5,6]. This clinical dilemma underscores a ‘residual renal risk’ in DKD [7,8]. Accumulating evidence suggests that, independent of traditional glucolipotoxicity, low-grade inflammatory responses and disruptions in immune microenvironment homeostasis within the local renal tissue may contribute to DKD fibrotic progression [9,10].

In recent years, the role of the exposome in the pathogenesis of metabolic diseases has garnered increasing attention within global public health [11,12]. Volatile organic compounds (VOCs), ubiquitous environmental pollutants generated by industrialization, can cause profound disturbances to endocrine and metabolic homeostasis [13,14]. Xylene is among the most frequently detected typical VOCs [15]. While previous studies have primarily focused on the neurotoxicity and respiratory associations of xylene [16,17], several cross-sectional investigations suggest that VOC exposure might correlate with declining renal function in the general population [18,19]. Nevertheless, whether xylene exposure constitutes an independent risk factor for DKD, and whether it contributes to local immune microenvironmental remodeling, remains largely unelucidated.

We hypothesized that environmental xylene exposure might contribute to DKD progression by altering the local inflammatory microenvironment. To evaluate this, we first examined the clinical epidemiological association between urinary xylene metabolites and DKD prevalence. We then utilized bulk transcriptomic data to identify potential key genes and immune characteristics. These findings were subsequently explored using single-cell RNA sequencing to assess cellular heterogeneity. Lastly, network toxicology and molecular docking were applied to explore the structural basis of the putative ‘environmental exposure–target interaction–macrophage polarization’ axis in DKD.

## 2. Methods

### 2.1. Data collection

The overall study design and analytical workflow are illustrated in Figure 1. Baseline epidemiological data for 29,902 participants were extracted from the NHANES (2011–2016) database [20]. Following established clinical guidelines, DKD was defined as diabetes mellitus with chronic kidney disease (CKD), operationalized by an estimated glomerular filtration rate (eGFR) < 60 mL/min/1.73 m^2^ and/or a urine albumin-to-creatinine ratio (UACR) > 30 mg/g [3,21,22]. The exposure factors were defined as 23 urinary VOC metabolites quantified using UPLC-MS/MS [23]. After applying exclusion criteria for missing data, 2,622 valid samples were included for analysis (Figure 2(A)). The eGFR was calculated using the 2021 CKD-EPI equation [24]. The calculation formulas for key related indicators are as follows:

$$\text{UACR }\left(\text{(mg}/\text{g)}\right)=\left[\text{[Urinary albumin }\left(\mathrm{(\mu}g/\text{mL)}\right)/\text{Urinary creatinine }\left(\text{(mg}/\text{dL)]}\right)\right]\times 100$$

$$\text{eGFR}=142\times \text{min(Scr/}\kappa \text{, }{\text{1)}}^{\alpha }\times \text{max(Scr/}\kappa \text{, }{\text{1)}}^{-1.200}\times 0{\text{.9938}}^{\text{Age}}\times \text{(1.012 if female)}$$

**Figure 1.**
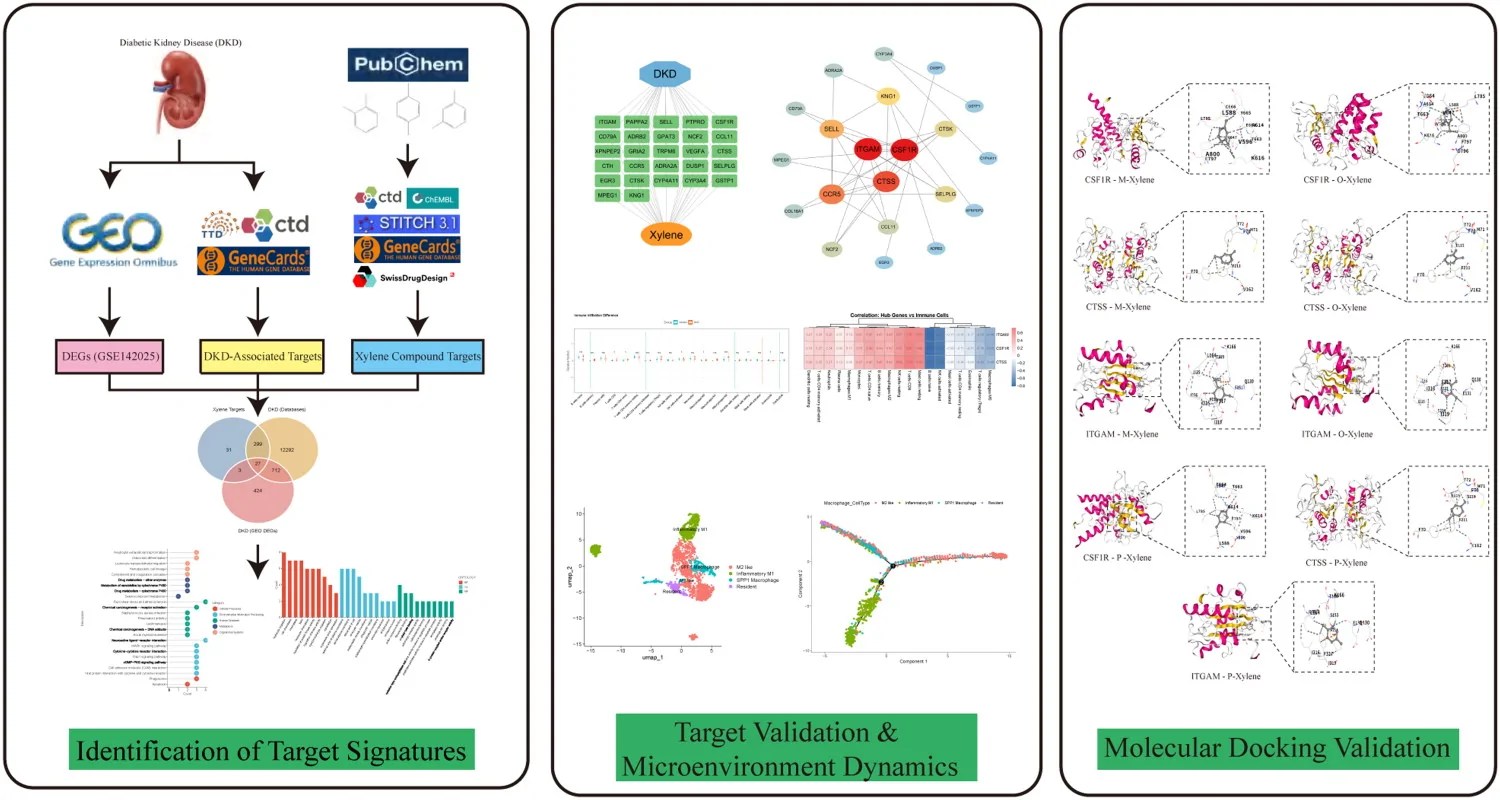
Schematic diagram of the overall study design and analytical workflow. The comprehensive analysis comprises three primary phases. (1) Identification of target signatures: Xylene-associated targets were mined from multiple public toxicological databases and intersected with diabetic kidney disease (dKD)-related targets and differentially expressed genes (DEGs) derived from the GEO dataset to identify core shared targets. (2) Target validation & microenvironment dynamics: A protein–protein interaction (PPI) network was constructed to screen for critical hub genes (ITGAM, CSF1R, CTSS). Subsequently, bulk immune infiltration analysis and single-cell RNA sequencing were utilized to delineate macrophage heterogeneity, polarization states, and pseudotime developmental trajectories within the DKD microenvironment. (3) Molecular docking validation: Computational molecular docking was performed to simulate and verify the direct binding affinities and interaction sites between three xylene isomers (m-xylene, o-xylene, and p-xylene) and the identified hub proteins.

**Figure 2.**
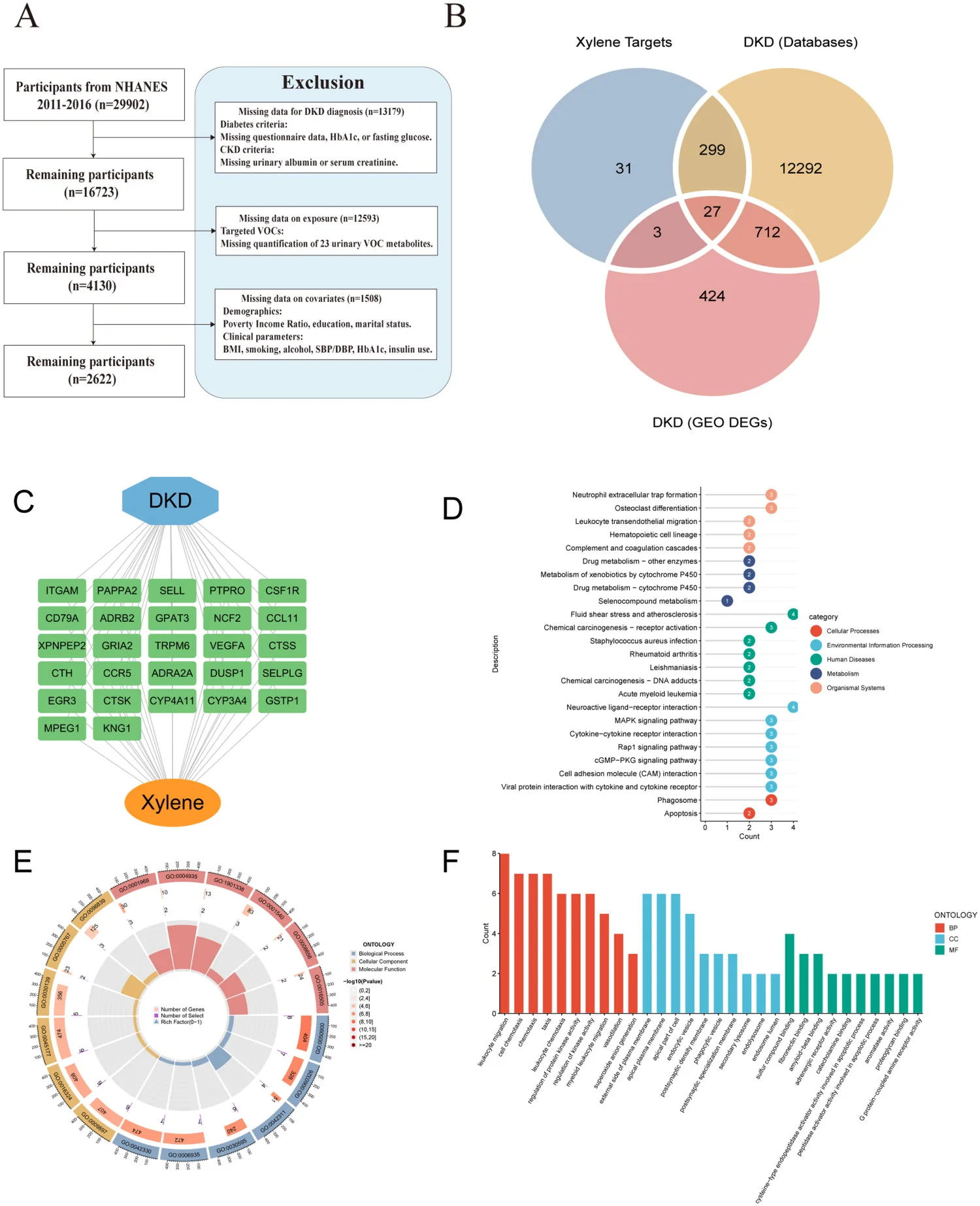
Identification of shared molecular targets between xylene exposure and DKD. (A) Flowchart detailing the selection of the NHANES 2011–2016 study population. (B) Venn diagram illustrating the intersection of xylene-associated targets, DKD disease database targets, and differentially expressed genes (DEGs) from the GEO dataset (GSE142025), yielding 27 core targets. (C) The compound-disease-target multidimensional regulatory network. (D) Bubble plot detailing the Kyoto Encyclopedia of Genes and Genomes (KEGG) pathway enrichment analysis of the core targets. (E) Circular plot and (F) bar chart representing the Gene Ontology (GO) enrichment analysis, highlighting biological processes (BP), cellular components (CC), and molecular functions (MF) predominantly associated with immune responses and leukocyte migration.

### 2.2. Bulk transcriptomic analysis

To investigate clinical tissue relevance, bulk transcriptomic data of human normal and DKD renal tissues (GSE142025) were acquired from the NCBI GEO database [25]. Differentially expressed genes (DEGs) were calculated using the limma package in R, with significance thresholds set at |log2FC| ≥ 1 and adjusted *p* < 0.05.

### 2.3. Identification of key genes

Based on exposure biomarker standards [26], the specific positive metabolite (DPMA) was mapped back to its parent xylene isomers. Target databases (PubChem, ChEMBL, CTD) were queried for potential xylene-binding targets [27–29]. Simultaneously, disease-related targets for DKD were compiled from the TTD, CTD, and GeneCards databases. Venn intersection analysis was applied across compound targets, disease targets, and GEO DEGs to pinpoint core regulatory targets. Hub genes were subsequently identified by constructing a Protein-Protein Interaction (PPI) network *via* the STRING database (v12.0) [30] and applying topological degree centrality sorting using Cytoscape (v3.9.1) [31,32]. To independently validate their diagnostic value and mitigate potential literature annotation bias, receiver operating characteristic (ROC) curve analysis was subsequently performed.

### 2.4. Functional enrichment analysis

The clusterProfiler package in R [33] was utilized to perform Gene Ontology (GO) annotation and Kyoto Encyclopedia of Genes and Genomes (KEGG) signaling pathway enrichment analysis on the core targets. The significance threshold was set at *p* < 0.05 to characterize the macroscopic functional tendencies of the identified genes.

### 2.5. Immune infiltration analysis

To investigate the correlation between target expression and microenvironmental remodeling, the CIBERSORT deconvolution algorithm [34] was applied to the bulk transcriptomic data (GSE142025), which comprised 9 DKD and 22 normal control renal tissue samples. The relative infiltration abundance of 22 immune cell types was estimated using the LM22 leukocyte signature matrix with 1,000 permutations. Immune cell populations with zero abundance across all samples were excluded from subsequent analyses. Subsequently, Spearman correlation analysis was conducted to examine associations between hub gene expression and immune cell populations.

### 2.6. Construction of interaction model

High-resolution 3D crystal structures of the proteins encoded by the hub genes were retrieved from the Protein Data Bank (PDB). Molecular docking was performed using the CB-Dock2 platform equipped with the AutoDock Vina engine [35,36]. The thermodynamic binding free energy (Vina Score) was calculated to assess the theoretical binding affinity between xylene ligands and receptor proteins.

### 2.7. Single-Cell RNA sequencing analysis

To validate the macrophage-related findings from bulk transcriptomic analyses, an independent human kidney Single-cell RNA sequencing dataset (GSE209781) was analyzed using the Seurat package in R. Quality control was performed to remove low-quality cells, retaining cells with >300 detected genes, >1,000 UMIs, and <20% mitochondrial gene content. Potential doublets were identified and removed using DoubletFinder. Gene expression data were normalized using the LogNormalize method, and the top 2,000 highly variable genes were selected using the vst method. Batch effects among samples were corrected using Harmony. PCA was performed, and the top principal components (PCs 1–20 for the global dataset and PCs 1–15 for the immune subset) were used for UMAP visualization and clustering analysis. Cell clusters were identified using a graph-based clustering approach (resolution = 0.5), and marker genes were detected using the MAST algorithm (logfc.threshold = 0.25). Cell types were annotated according to established canonical markers. Pseudotime trajectory analysis was subsequently conducted using Monocle 2 with DDRTree dimensionality reduction to infer macrophage differentiation trajectories and characterize the dynamic expression patterns of hub genes.

### 2.8. Statistical analysis

All statistical analyses were conducted in R (version 4.2.2). For the NHANES dataset, sampling weights were incorporated using the survey package [37]. Continuous urinary VOC metabolite concentrations were log-transformed prior to analysis. Multivariable logistic regression models were used to evaluate the associations between urinary VOC metabolites and diabetic kidney disease (DKD), with results reported as odds ratios (ORs) and 95% confidence intervals (CIs). Four progressively adjusted models were constructed. Model 1 was adjusted for urinary creatinine. Model 2 was additionally adjusted for age, sex, race/ethnicity, poverty income ratio (PIR), education level, and marital status. Model 3 was further adjusted for body mass index (BMI), smoking status, alcohol consumption, systolic blood pressure (SBP), diastolic blood pressure (DBP), glycated hemoglobin (HbA1c), and insulin use. To account for potential co-exposure effects among VOC metabolites, Model 4 additionally adjusted for the remaining 22 urinary VOC metabolites. To mitigate potential sparse-data bias resulting from the low detection frequency of DPMA, Firth’s penalized logistic regression was employed to calculate the odds ratios (ORs) and 95% confidence intervals (CIs). A two-sided *p* value < 0.05 and an FDR < 0.05 were considered statistically significant.

## 3. Results

### 3.1. Clinical relevance analysis

Baseline clinical and demographic characteristics of the study population are summarized in Table 1. Multivariable logistic regression analysis of the NHANES cohort (Table 2) demonstrated that the levels of the xylene-specific urinary metabolite DPMA were positively associated with the odds of DKD. Following progressive adjustment for baseline demographic characteristics (Model 2) and clinical parameters (including smoking, BMI, blood pressure, HbA1c, and insulin use in Model 3), this association remained significant (Model 3: OR = 4.03, 95% CI: 1.80–9.00, *p* = 0.002; FDR = 0.036). Furthermore, a new sensitivity analysis adjusting for the co-exposure of the other 22 urinary VOC metabolites confirmed that this independent association persisted (Model 4: OR = 3.91, 95% CI: 1.68–9.10, *p* = 0.003), and remained statistically significant in Firth’s penalized logistic regression. The multivariable logistic regression results for all urinary VOC metabolites, as well as the Firth regression results, are presented in Supplementary Table S1.

**Table 1.** Baseline clinical and demographic characteristics of the study population from NHANES.

Characteristic	Overall (*N* = 2622)	Control (*N* = 2447)	DKD (*N* = 175)	*p*-value
DPMA detection status				0.011
≥LOD	9 (0.3%)	6 (0.2%)	3 (1.7%)	
<LOD	2,613 (99.7%)	2,441 (99.8%)	172 (98.3%)	
Age	46.0 (32.0, 61.0)	45.0 (31.0, 60.0)	66.0 (56.0, 74.0)	<0.001
Sex				0.043
Male	1,357 (51.8%)	1,253 (51.2%)	104 (59.4%)	
Female	1,265 (48.2%)	1,194 (48.8%)	71 (40.6%)	
Race				0.004
Mexican American	318 (12.1%)	289 (11.8%)	29 (16.6%)	
Other Hispanic	312 (11.9%)	295 (12.1%)	17 (9.7%)	
Non-Hispanic White	1,003 (38.3%)	942 (38.5%)	61 (34.9%)	
Non-Hispanic Black	591 (22.5%)	538 (22%)	53 (30.3%)	
Non-Hispanic Asian	319 (12.2%)	304 (12.4%)	15 (8.6%)	
Other race	79 (3%)	79 (3.2%)	0 (0.0%)	
Poverty income ratio	2.1 (1.1, 4.3)	2.2 (1.1, 4.4)	1.4 (0.9, 2.3)	<0.001
Education				<0.001
<9th grade	221 (8.4%)	182 (7.4%)	39 (22.3%)	
9–11th grade	296 (11.3%)	262 (10.7%)	34 (19.4%)	
High school grad	544 (20.7%)	504 (20.6%)	40 (22.9%)	
Some college	793 (30.2%)	755 (30.9%)	38 (21.7%)	
College grade	768 (29.3%)	744 (30.4%)	24 (13.7%)	
Marital status				<0.001
Married/Partnered	1,543 (58.8%)	1,447 (59.1%)	96 (54.9%)	
Widowed/Divorced	492 (18.8%)	431 (17.6%)	61 (34.9%)	
Never married	587 (22.4%)	569 (23.3%)	18 (10.3%)	
BMI	27.9 (24.3, 32.7)	27.7 (24.2, 32.4)	30.9 (26.8, 36.9)	<0.001
Smoking status				<0.001
No	1,493 (56.9%)	1,417 (57.9%)	76 (43.4%)	
Yes	1,129 (43.1%)	1,030 (42.1%)	99 (56.6%)	
Alcohol				0.073
No	667 (25.4%)	612 (25%)	55 (31.4%)	
Yes	1,955 (74.6%)	1,835 (75%)	120 (68.6%)	
Systolic BP (mmHg)	120.0 (110.0, 132.0)	120.0 (110.0, 130.0)	136.0 (124.0, 150.0)	<0.001
Diastolic BP (mmHg)	70.0 (64.0, 78.0)	70.0 (64.0, 78.0)	68.0 (58.0, 78.0)	0.037
eGFR	99.1 (84.0, 112.0)	100.1 (86.1, 113.0)	63.2 (49.1, 95.9)	<0.001
UACR	6.5 (4.4, 11.1)	6.2 (4.3, 10.0)	58.7 (30.9, 224.9)	<0.001

**Table 2.** Multivariable logistic regression analyses of the association between urinary DPMA levels and diabetic kidney disease.

Characteristic	Model 1			Model 2			Model 3			Model 4		
	OR	95% CI	*p*-value	OR	95% CI	*p*-value	OR	95% CI	*p*-value	OR	95% CI	*p*-value
DPMA	4.46	2.46, 8.09	<0.001	3.05	1.47, 6.29	0.004	4.03	1.80, 9.00	0.002	3.91	1.68, 9.10	0.003

Model 1: Adjusted for urine creatinine.

Model 2: Adjusted for urine creatinine, age, sex, race, PIR, education, and marital status.

Model 3: Adjusted for urine creatinine, age, sex, Race, PIR, education, marital status, BMI, smoke, alcohol, SBP, DBP, HbA1c, and insulin use.

Model 4: Adjusted for urine creatinine and the other 22 urinary VOC metabolites.

### 3.2. Identification of differentially expressed genes and key targets

Toxicokinetic studies indicate that urinary DPMA serves as a specific biomarker for xylene isomers [26]. By integrating compound databases, DKD disease databases, and DEGs from the GEO bulk transcriptomic dataset (GSE142025) (Table 3), Venn intersection algorithms identified 27 core targets mediating potential interactions between xylene exposure and DKD (Figure 2(B)). A multidimensional regulatory network model was constructed based on these targets (Figure 2(C)).

**Table 3.** Summary of compound structures, disease targets, and differentially expressed genes retrieved from public databases.

Data category	Data source	Specific search items	Obtained results	Targets after deduplication	Remarks
Compound structure	PubChem	O-Xylene	CC1 = CC = CC = C1C	—	—
	PubChem	P-Xylene	CC1 = CC = C(C = C1)C	—	—
	PubChem	M-Xylene	CC1 = CC(=CC = C1)C	—	—
Compound targets	ChEMBL	O-Xylene (81) P-Xylene (83) M-Xylene (95)	259	224	None
	CTD	O-Xylene (52) P-Xylene (11) M-Xylene (36)	99	84	None
	GeneCards	O-Xylene (6) P-Xylene (5) M-Xylene (57)	68	64	Score > 0.5
	STITCH	O-Xylene (9) P-Xylene (3) M-Xylene (7)	19	7	None
	SwissTargetPrediction	O-Xylene (1) P-Xylene (2) M-Xylene (1)	4	2	probability > 0
	Total: 60				
Disease targets	TTD	DKD (29) DN (28)	57	29	None
	CTD	DKD (11668) DN (11663)	23331	11663	Inference score > 10
	GeneCards	DKD (5994) DN (858)	6852	5998	Score > 10
	Total: 13330				
Transcriptome DEGs	GEO (GSE142025)	Differential Analysis	1166	1166	|log2FC| ≥ 1 & *p*_adj_ < 0.05

### 3.3. Functional enrichment analysis

GO and KEGG enrichment analyses of the 27 core targets highlighted consistent pathological pathways (Figure 2(D–F), with detailed high-resolution results provided in Supplementary Table S2). Biological processes were primarily enriched in local immune reactions, including leukocyte migration and chemotaxis. At the pathway level, alongside conventional exogenous drug metabolism, there was an enrichment in classic immune pathways such as ‘Neutrophil extracellular trap formation,’ ‘Cytokine-cytokine receptor interaction,’ and ‘Phagosome,’ supporting the potential involvement of the immune microenvironment.

### 3.4. Identification and clinical validation of candidate hub genes

Topological analysis of the PPI network identified ITGAM, CSF1R, and CTSS as the top candidate hub genes based on degree centrality (Figure 3(A)). ROC analysis in the GSE142025 dataset showed that all three genes could distinguish DKD from normal kidney tissues, with AUC values of 0.844 for CSF1R, 0.807 for ITGAM, and 0.782 for CTSS (all *p* < 0.05; Supplementary Figure S1). These results suggest that the identified hub genes may be associated with DKD-related transcriptional alterations.

**Figure 3.**
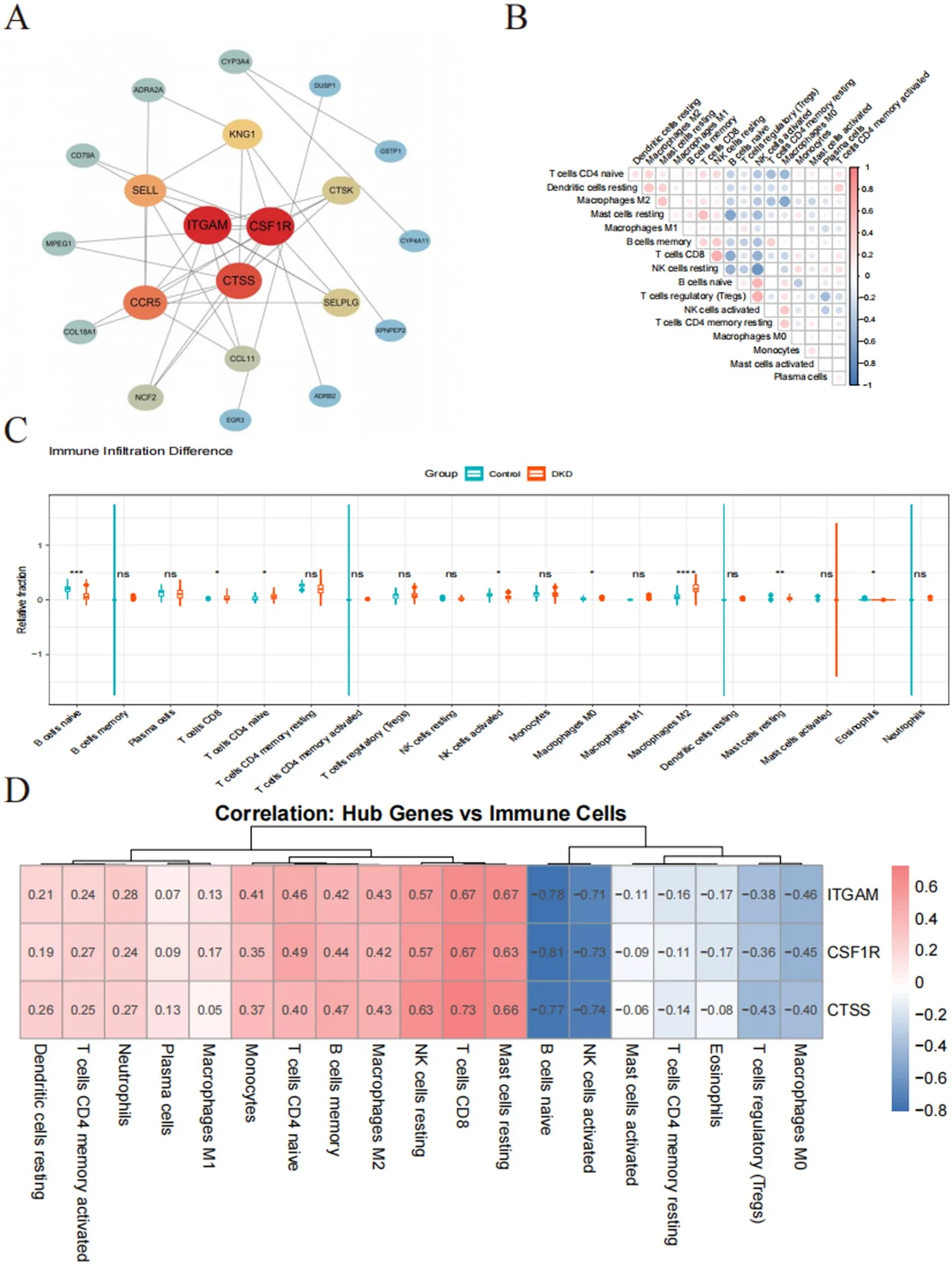
Hub gene identification and bulk immune microenvironment characterization. (A) Protein–protein interaction (PPI) network of the 27 core targets; node color and size indicate topological degree centrality, identifying ITGAM, CSF1R, and CTSS as primary hub genes. (B) Correlation matrix depicting the interplay among 22 immune cell types within the renal microenvironment, evaluated *via* CIBERSORT deconvolution. (C) Boxplot comparing the relative infiltration fractions of immune cells between normal controls and DKD tissues. Statistical significance is denoted as *p < 0.05, **p < 0.01, ***p < 0.001. (D) Heatmap demonstrating Spearman correlations between the expression levels of the three hub genes (ITGAM, CSF1R, CTSS) and various immune cell populations, revealing strong positive associations with M2 macrophages.

### 3.5. Immune microenvironment characteristics

Bulk immune deconvolution *via* CIBERSORT revealed differences in immune cell distributions between DKD and control tissues (Figure 3(B,C)). Specifically, DKD kidneys exhibited a higher infiltration of M2-like macrophages associated with fibrotic remodeling and CD8^+^ T cells. Spearman correlation analysis (Figure 3(D)) demonstrated that the expression levels of the ITGAM, CSF1R, and CTSS hub genes positively correlated with the abundance of M2-like macrophages (R0.42–0.47).

### 3.6. Molecular docking validation

To explore potential molecular interaction mechanisms, docking simulations evaluated spatial binding affinities (Figure 4). The simulations indicated that xylene isomers might form stable conformations within the putative ligand-binding pockets of these receptors, with binding free energies ranging from −4.7 to −5.9 kcal/mol (Table 4). These computational predictions are compatible with the hypothesis that xylene may interact with these receptors; however, the modest binding energies underscore the need for independent biophysical validation.

**Figure 4.**
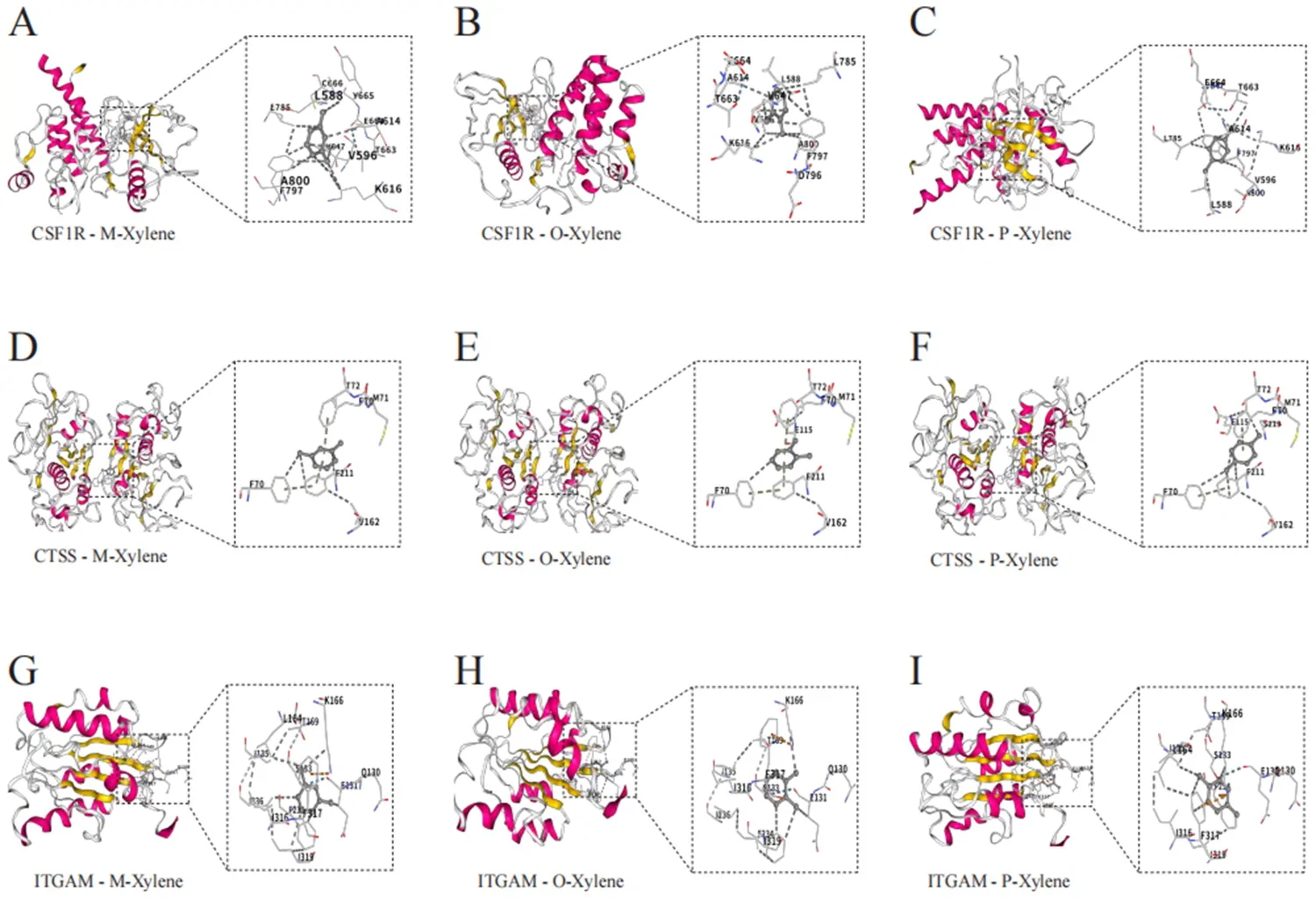
Molecular docking simulations of xylene isomers with immune receptor pockets. High-resolution 3D spatial conformations and local binding interactions of three xylene isomers (m-Xylene, o-Xylene, p-Xylene) with the putative hydrophobic binding pockets of the hub proteins. (A–C) Docking models for CSF1R. (D–F) Docking models for CTSS. (G–I) Docking models for ITGAM. Dashed lines in the magnified views indicate specific amino acid residue interactions. Thermodynamic binding scores are detailed in Table 4.

**Table 4.** Thermodynamic parameters and binding affinity evaluation of molecular docking between xylene isomers and target proteins.

Target protein (PDB ID)	Ligand (compound)	Vina score (kcal/mol)	Cavity volume (Å³)	Grid center (*x*, *y*, *z*)	Grid size (*x*, *y*, *z*)
ITGAM (8CE6)	o-Xylene	−4.7	384	0, 25, 44	16, 16, 16
	m-Xylene	−4.9	384	0, 25, 44	16, 16, 16
	p-Xylene	−4.9	384	0, 25, 44	16, 16, 16
CSF1R (8CGC)	o-Xylene	−5.8	424	48, 43, −7	16, 16, 16
	m-Xylene	−5.9	424	48, 43, −7	16, 16, 16
	p-Xylene	−5.8	424	48, 43, −7	16, 16, 16
CTSS (4P6E)	o-Xylene	−5.5	1571	29, 31, 17	28, 23, 16
	m-Xylene	−5.5	1571	29, 31, 17	28, 23, 16
	p-Xylene	−5.5	1571	29, 31, 17	28, 23, 16

### 3.7. Validation using single-cell RNA sequencing

To further examine the macrophage alterations suggested by the bulk data, we analyzed the Single-cell RNA sequencing dataset (GSE209781). UMAP clustering annotated major renal cell types (Figure 5(A,B)). Sub-clustering of macrophages identified distinct functional states: resident, inflammatory M1, SPP1+ macrophages, and M2-like macrophages. Consistent with the bulk findings, the M2-like and SPP1+ pro-fibrotic subsets appeared expanded in DKD tissues (Figure 5(C–E)). Moreover, the identified hub genes (ITGAM, CSF1R, and CTSS) exhibited elevated expression predominantly within these specific macrophage clusters (Figure 5(F,G)).

**Figure 5.**
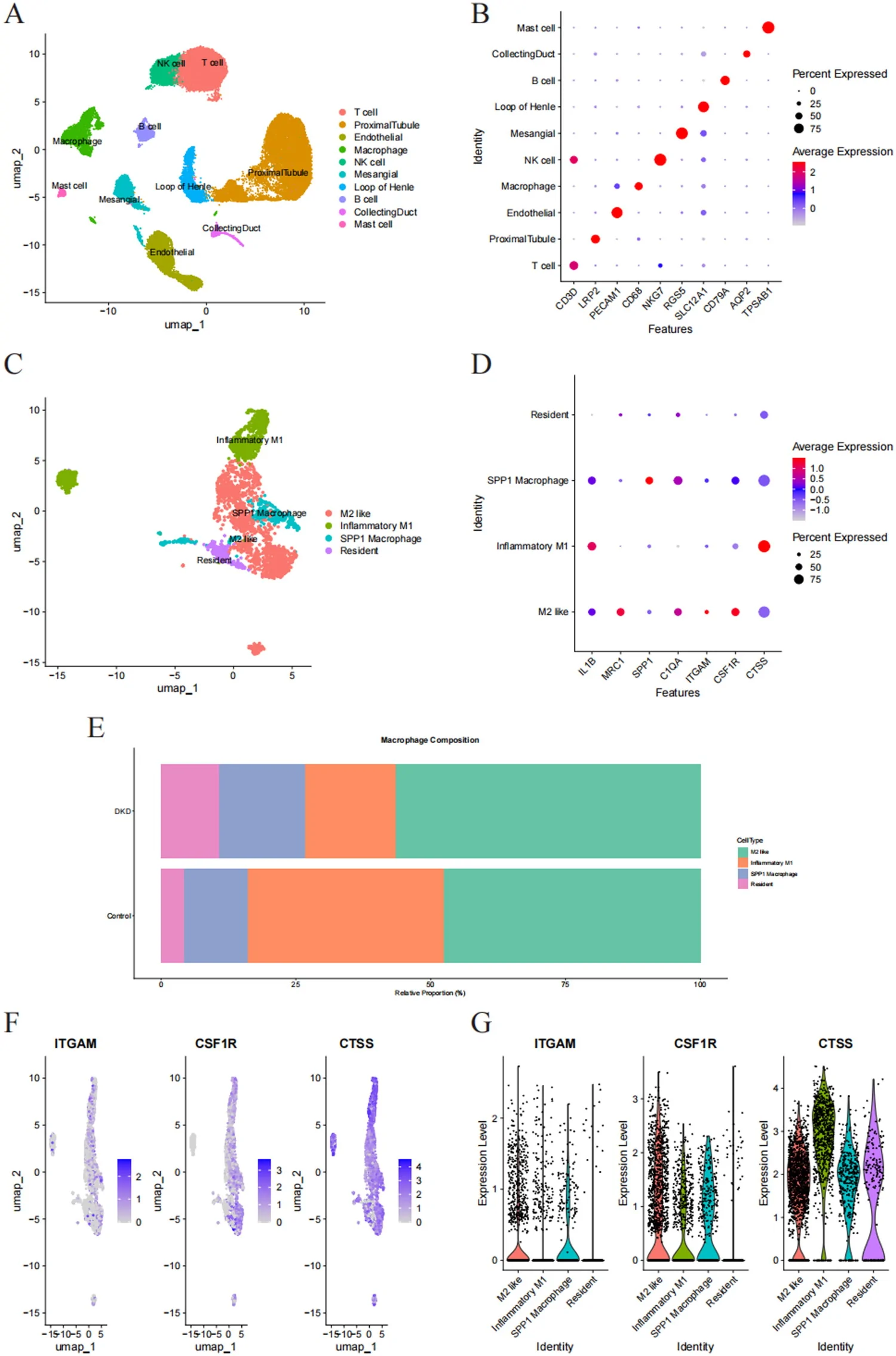
Single-cell RNA sequencing reveals the expansion of pro-fibrotic macrophages in DKD. (A) Uniform manifold approximation and projection (UMAP) of major renal cell lineages derived from the GSE209781 dataset. (B) Dot plot showing the expression of canonical marker genes used for cell type annotation. (C) UMAP sub-clustering of the macrophage lineage into four distinct states: resident, inflammatory M1, SPP1+ macrophage, and M2-like macrophage. (D) Dot plot of specific marker genes defining the macrophage sub-clusters. (E) Bar plot quantifying the relative proportion of macrophage sub-clusters in control versus DKD conditions, highlighting the expansion of M2-like and SPP1+ subsets in DKD. (F) UMAP feature plots and (G) Violin plots illustrating the spatial distribution and expression intensity of the hub genes (ITGAM, CSF1R, CTSS), which are highly enriched in the pro-fibrotic subsets.

Pseudotime trajectory analysis (Figure 6) modeled the developmental dynamics, revealing a continuous path from resident macrophages toward terminally differentiated states enriched in DKD. The expression of CSF1R, CTSS, and established polarization markers (IL1B, MRC1, SPP1) increased along this pseudotime axis, consistent with the observations derived from bulk RNA-seq at a single-cell resolution.

**Figure 6.**
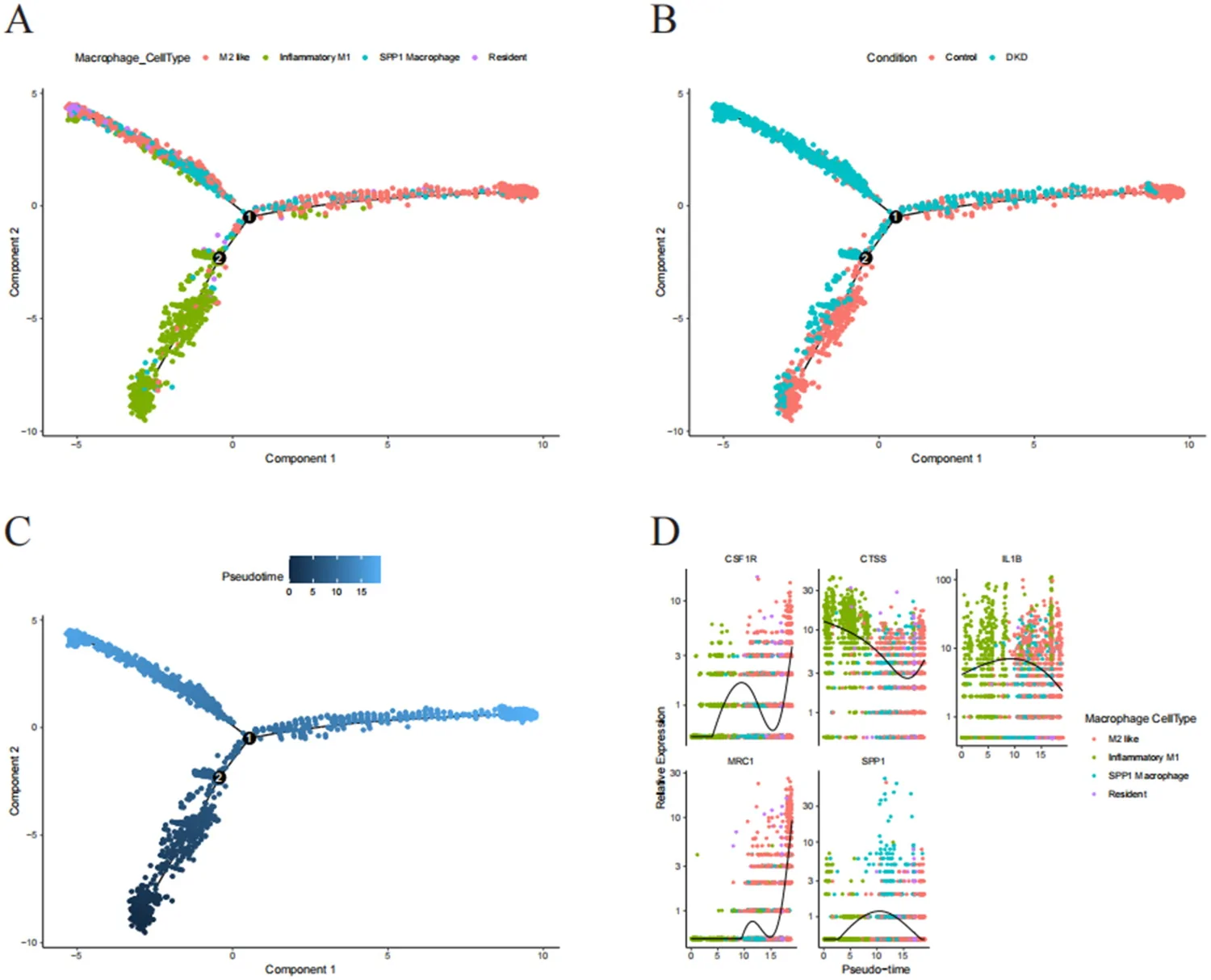
Pseudotime trajectory analysis of macrophage developmental dynamics. (A) Monocle-based developmental trajectory mapped and colored by macrophage sub-cluster identities. (B) The identical trajectory mapped by disease condition (control vs. DKD), demonstrating a shift toward terminal states in DKD. (C) Pseudotime progression gradient from root (dark blue) to terminal differentiation (light blue). (D) Dynamic gene expression profiles along the pseudotime axis, showing the progressive upregulation of hub genes (CSF1R, CTSS) alongside established pro-fibrotic polarization markers (IL1B, MRC1, SPP1).

## 4. Discussion

Using a multi-omics and exposomics approach, this cross-sectional study identified an independent epidemiological association between environmental xylene exposure and increased DKD prevalence. Through bulk transcriptomic screening and single-cell RNA sequencing analysis, ITGAM, CSF1R, and CTSS were identified as hub genes enriched in immune-related pathways. While the cross-sectional design precludes causal inference, these findings generate the hypothesis that xylene may interact with these specific macrophage receptors, potentially promoting M2-like macrophage polarization and thereby contributing to the residual inflammatory risk in DKD [38,39]. However, the observed association should be interpreted with caution given the small number of participants with detectable DPMA levels (*n* = 9), the wide confidence interval, and the potential for residual confounding.

While previous environmental nephropathy studies have highlighted heavy metals [40] and broader air pollution [41] as risk factors, recent NHANES analyses have begun linking VOCs to metabolic disturbances [18,42] and chronic kidney disease [19]. Building on this, our study specifically targeted the vulnerable diabetic population, finding that xylene exposure is independently associated with higher odds of DKD.

Previous studies have indicated that macrophage recruitment and phenotypic polarization play pivotal roles in DKD progression [43]. Excessive polarization toward the M2 phenotype may accelerate interstitial fibrosis through the secretion of transforming growth factor-beta (TGF-β) [44,45]. Because our epidemiological and transcriptomic datasets are independent, our findings do not imply that xylene initiates this immune infiltration. Rather, we hypothesize that environmental xylene exposure may act as a superimposed environmental stressor that intersects with these established pro-fibrotic pathways. Specifically, xylene might interact with specific macrophage receptors to exacerbate the existing M2-driven residual inflammatory risk in DKD. This concept aligns with emerging evidence in environmental immunotoxicology indicating that exogenous pollutants can modulate macrophage phenotypes and aggravate tissue fibrosis [46,47]. While the initial identification of these candidate genes relied on PPI network centrality, supplementary ROC analysis provided additional support for their potential relevance to DKD, with AUC values ranging from 0.782 to 0.844. Existing evidence suggests that ITGAM (CD11b) participates in monocyte/macrophage transendothelial migration [48], CSF1R contributes to the regulation of pro-fibrotic M2 macrophage polarization [49,50], and CTSS (Cathepsin S) is involved in extracellular matrix remodeling [51]. Our in silico molecular docking analysis indicates that the hydrophobic structure of xylene permits moderate binding to these receptor cavities (binding free energies: −4.7 to −5.9 kcal/mol). These computational predictions generate the hypothesis that xylene could potentially interfere with receptor signaling; however, the modest binding energies indicate that further biophysical validation is required to establish biologically meaningful interactions.

We incorporated single-cell RNA sequencing data (GSE209781) to further evaluate our bulk transcriptomic inferences at a single-cell resolution. While bulk RNA-seq deconvolution indicated a general enrichment of M2 macrophages correlating with hub gene expression, it lacked cellular resolution. The single-cell RNA sequencing analysis supported these findings by revealing that the M2-like and SPP1+ macrophage subpopulations are expanded in the DKD microenvironment. Expression distribution analyses further revealed that ITGAM, CSF1R, and CTSS were highly localized within these pro-fibrotic subsets. Pseudotime trajectory analysis aligned with this concept by mapping the developmental progression toward the SPP1+ fibrotic state, providing single-cell resolution support consistent with the bulk-derived hub gene findings.

Clinically, these findings provide insights into the management of ‘residual renal risk.’ Routine DKD management currently places little emphasis on environmental toxicant exposures. The incorporation of VOC exposure assessments – such as occupational hazards or exhaust fumes – into the risk stratification of patients with diabetes may hold preventive value. Additionally, the identified targets, particularly CSF1R, present potential avenues for intervention. Small-molecule inhibitors targeting CSF1R have shown promise in experimental tumor immunotherapy and fibrotic diseases [52]. Exploring their adjunctive therapeutic potential to modulate macrophage polarization in DKD presents an area for future investigation.

This study benefits from a multi-tiered approach that links population epidemiology (NHANES) with transcriptomic screening, structural mechanism prediction, and single-cell resolution analysis. However, several limitations must be acknowledged. First, the cross-sectional nature of the NHANES data prevents the assessment of longitudinal baseline renal trajectories, thereby limiting strict causal inference [20]. Furthermore, although we adjusted for key clinical covariates including glycemic control and total measured VOC burden, residual confounding from unmeasured factors – such as specific diabetes duration and the exact use of novel nephroprotective agents (e.g., SGLT2 inhibitors and RAAS blockers) – cannot be entirely excluded due to database limitations. Second, the low detection frequency of urinary DPMA (*n* = 9) creates susceptibility to sparse-data bias and wide confidence intervals. Although we employed Firth’s penalized logistic regression to statistically mitigate this bias and stabilize the estimates across multiple rigorously adjusted models, the results must still be interpreted with caution. Furthermore, DPMA has a relatively short biological half-life, which may introduce non-differential misclassification bias, primarily reflecting recent rather than cumulative exposure. Therefore, the population-level findings are considered hypothesis-generating and exploratory. Future prospective cohorts with repeated exposure assessments and larger sample sizes are required to confirm these robust, yet preliminary, associations. Third, the transcriptomic and single-cell datasets were generated from independent cohorts and were not linked to the NHANES participants at the individual level. Consequently, the multi-layered analyses provide complementary evidence across different data modalities but do not constitute a directly integrated multi-omics analysis. Fourth, the multiple VOC metabolites tested without formal correction for multiplicity should be considered when interpreting the reported associations. Lastly, the proposed ‘receptor binding-macrophage polarization’ axis derived from multi-omics data is fundamentally predictive and warrants further rigorous functional validation through *in vivo* and *in vitro* experiments [53].

Future research should prioritize longitudinal exposome cohorts to track the temporal relationship between VOCs and renal decline more definitively. Furthermore, basic experimental studies, such as utilizing macrophage-specific knockout murine models exposed to xylene, are needed to biologically verify the signaling perturbations suggested by our single-cell and computational analyses.

## 5. Conclusion

Through epidemiological investigation and multi-omics systems biology, this study identified an association between environmental xylene exposure and increased DKD odds. Supported by bulk and single-cell transcriptomic data, we hypothesize that xylene may interact with specific macrophage receptors (ITGAM, CSF1R, CTSS), potentially contributing to M2-like macrophage polarization and fibrotic microenvironment remodeling. These findings, while associational in nature, expand the understanding of environmental immunotoxicology in DKD and generate novel hypotheses regarding potential diagnostic and therapeutic targets for residual inflammatory risk that warrant future experimental validation.

## Supplementary Material

Supplementary Table S1.docx

Supplementary Figure S1.tif

Supplementary Table_S2.xlsx

## Data Availability

Publicly available datasets were analyzed in this study. The epidemiological data can be found in the NHANES database (https://www.cdc.gov/nchs/nhanes/). The bulk and single-cell transcriptomic data can be found in the NCBI GEO database (https://www.ncbi.nlm.nih.gov/geo/) under accession numbers GSE142025 and GSE209781.
