# Supplementary material for: Association of environmental xylene exposure with diabetic kidney disease: a cross-sectional analysis and multi-omics evaluation of immune microenvironment mechanisms
Source: Ren Fail. 2026 Oct 7;48(1):2738974. doi: 10.1080/0886022X.2026.2738974 (PMC13647346; doi:10.1080/0886022X.2026.2738974)
Supplement: Supplementary file 1 [file IRNF_A_2738974_SM2988.docx]

**Supplementary Table S1-1.** Association between urinary DPMA and the risk of Diabetic Kidney Disease (DKD) assessed by Firth’s penalized logistic regression.

| **Characteristic** | **Model 1** | | | **Model 2** | | | **Model 3** | | | **Model 4** | | |
| --- | --- | --- | --- | --- | --- | --- | --- | --- | --- | --- | --- | --- |
|  | **OR** | **95% CI** | **p-value** | **OR** | **95% CI** | **p-value** | **OR** | **95% CI** | **p-value** | **OR** | **95% CI** | **p-value** |
| DPMA | 4.16 | 2.09, 9.90 | <0.001 | 2.72 | 1.25, 7.46 | 0.009 | 3.35 | 1.38, 12.3 | 0.006 | 3.82 | 1.70, 9.39 | <0.001 |

Model 1: Adjusted for Urine Creatinine.

Model 2: Adjusted for Urine Creatinine, Age, Gender, Race, PIR, Education, and Marital Status.

Model 3: Adjusted for Urine Creatinine, Age, Gender, Race, PIR, Education, Marital Status, BMI, Smoke, Alcohol, SBP, DBP, HbA1c, and Insulin Use.

Model 4: Adjusted for Urine Creatinine and the other 22 urinary VOC metabolites.

**Supplementary Table S1-2.** Multivariable logistic regression analyses of the associations between urinary volatile organic compound (VOC) metabolites and diabetic kidney disease

| **Characteristic** | **Model 1** | | | **Model 2** | | | **Model 3** | | | |
| --- | --- | --- | --- | --- | --- | --- | --- | --- | --- | --- |
|  | **OR** | **95% CI** | **p-value** | **OR** | **95% CI** | **p-value** | **OR** | **95% CI** | **p-value** | **FDR** |
| DPMA | 4.46 | 2.46, 8.09 | <0.001 | 3.05 | 1.47, 6.29 | 0.004 | 4.03 | 1.80, 9.00 | 0.002 | 0.036 |
| 2,2-DCV | 1.03 | 0.73, 1.44 | 0.900 | 0.94 | 0.65, 1.35 | 0.700 | 0.91 | 0.61, 1.37 | 0.600 | 0.979 |
| 2-MHA | 0.84 | 0.70, 1.01 | 0.057 | 0.87 | 0.73, 1.05 | 0.150 | 0.96 | 0.76, 1.21 | 0.700 | 0.979 |
| 3,4-MHA | 0.92 | 0.76, 1.13 | 0.400 | 0.89 | 0.73, 1.08 | 0.200 | 0.96 | 0.76, 1.20 | 0.700 | 0.979 |
| AAMA | 0.92 | 0.76, 1.12 | 0.400 | 0.94 | 0.77, 1.15 | 0.600 | 1.00 | 0.81, 1.25 | >0.900 | 0.979 |
| AMCC | 1.14 | 0.98, 1.33 | 0.083 | 1.03 | 0.85, 1.25 | 0.800 | 1.02 | 0.80, 1.30 | 0.900 | 0.979 |
| BMA | 1.06 | 0.90, 1.26 | 0.500 | 0.96 | 0.78, 1.18 | 0.700 | 1.02 | 0.83, 1.25 | 0.800 | 0.979 |
| BPMA | 0.91 | 0.77, 1.09 | 0.300 | 0.96 | 0.80, 1.16 | 0.700 | 0.97 | 0.77, 1.23 | 0.800 | 0.979 |
| CEMA | 1.18 | 0.93, 1.48 | 0.200 | 1.01 | 0.77, 1.33 | >0.900 | 0.95 | 0.66, 1.37 | 0.800 | 0.979 |
| CYMA | 0.98 | 0.90, 1.05 | 0.500 | 0.99 | 0.91, 1.07 | 0.800 | 0.99 | 0.90, 1.09 | 0.900 | 0.979 |
| DHBMA | 1.69 | 1.05, 2.72 | 0.032 | 1.02 | 0.63, 1.67 | >0.900 | 0.95 | 0.55, 1.63 | 0.800 | 0.979 |
| GAMA | 1.05 | 0.81, 1.37 | 0.700 | 1.11 | 0.77, 1.62 | 0.600 | 1.17 | 0.75, 1.83 | 0.500 | 0.979 |
| HEMA | 0.80 | 0.65, 0.98 | 0.035 | 0.82 | 0.68, 1.00 | 0.048 | 0.80 | 0.62, 1.02 | 0.073 | 0.843 |
| 2-HPMA | 0.93 | 0.80, 1.09 | 0.300 | 0.91 | 0.77, 1.09 | 0.300 | 0.93 | 0.74, 1.16 | 0.500 | 0.979 |
| 3-HPMA | 1.01 | 0.85, 1.20 | >0.900 | 1.01 | 0.86, 1.18 | >0.900 | 1.01 | 0.80, 1.28 | >0.900 | 0.979 |
| MA | 1.06 | 0.83, 1.36 | 0.600 | 1.05 | 0.81, 1.35 | 0.700 | 0.99 | 0.70, 1.39 | >0.900 | 0.979 |
| MHBMA-1 | 0.26 | 0.03, 2.62 | 0.200 | 0.35 | 0.05, 2.36 | 0.300 | 0.51 | 0.09, 2.76 | 0.400 | 0.979 |
| MHBMA-2 | 0.97 | 0.75, 1.25 | 0.800 | 0.93 | 0.71, 1.23 | 0.600 | 0.93 | 0.65, 1.34 | 0.700 | 0.979 |
| MHBMA-3 | 1.03 | 0.89, 1.19 | 0.700 | 0.96 | 0.81, 1.15 | 0.700 | 0.95 | 0.75, 1.20 | 0.600 | 0.979 |
| PHEMA | 1.13 | 0.93, 1.36 | 0.200 | 1.02 | 0.83, 1.24 | 0.900 | 1.04 | 0.84, 1.29 | 0.700 | 0.979 |
| PGA | 1.35 | 1.06, 1.72 | 0.016 | 1.14 | 0.89, 1.45 | 0.300 | 1.11 | 0.84, 1.49 | 0.400 | 0.979 |
| PMA | 1.06 | 0.85, 1.33 | 0.600 | 1.13 | 0.90, 1.43 | 0.300 | 1.12 | 0.84, 1.51 | 0.400 | 0.979 |
| HPMMA | 1.15 | 1.01, 1.30 | 0.030 | 1.05 | 0.90, 1.22 | 0.500 | 1.01 | 0.82, 1.25 | >0.900 | 0.979 |

Model 1: Adjusted for Urine Creatinine.

Model 2: Adjusted for Urine Creatinine, Age, Gender, Race, PIR, Education, and Marital Status.

Model 3: Adjusted for Urine Creatinine, Age, Gender, Race, PIR, Education, Marital Status, BMI, Smoke, Alcohol, SBP, DBP, HbA1c, and Insulin Use.
